# Quantitative Measurement of Melanoma Spread in Sentinel Lymph Nodes and Survival

**DOI:** 10.1371/journal.pmed.1001604

**Published:** 2014-02-18

**Authors:** Anja Ulmer, Klaus Dietz, Isabelle Hodak, Bernhard Polzer, Sebastian Scheitler, Murat Yildiz, Zbigniew Czyz, Petra Lehnert, Tanja Fehm, Christian Hafner, Stefan Schanz, Martin Röcken, Claus Garbe, Helmut Breuninger, Gerhard Fierlbeck, Christoph A. Klein

**Affiliations:** 1 Department of Dermatology, University of Tübingen, Tübingen, Germany; 2 Department of Medical Biometry, University of Tübingen, Tübingen, Germany; 3 Chair of Experimental Medicine and Therapy Research, Department of Pathology, University of Regensburg, Regensburg, Germany; 4 Project Group Personalized Tumor Therapy, Fraunhofer-Institut für Toxikologie und Experimentelle Medizin, Regensburg, Germany; 5 Department of Gynecology, University of Düsseldorf, Düsseldorf, Germany; 6 Department of Dermatology, University of Regensburg, Regensburg, Germany; University of Edinburgh, United Kingdom

## Abstract

**Background:**

Sentinel lymph node spread is a crucial factor in melanoma outcome. We aimed to define the impact of minimal cancer spread and of increasing numbers of disseminated cancer cells on melanoma-specific survival.

**Methods and Findings:**

We analyzed 1,834 sentinel nodes from 1,027 patients with ultrasound node-negative melanoma who underwent sentinel node biopsy between February 8, 2000, and June 19, 2008, by histopathology including immunohistochemistry and quantitative immunocytology. For immunocytology we recorded the number of disseminated cancer cells (DCCs) per million lymph node cells (DCC density [DCCD]) after disaggregation and immunostaining for the melanocytic marker gp100. None of the control lymph nodes from non-melanoma patients (*n = *52) harbored gp100-positive cells. We analyzed gp100-positive cells from melanoma patients by comparative genomic hybridization and found, in 45 of 46 patients tested, gp100-positive cells displaying genomic alterations. At a median follow-up of 49 mo (range 3–123 mo), 138 patients (13.4%) had died from melanoma. Increased DCCD was associated with increased risk for death due to melanoma (univariable analysis; *p<*0.001; hazard ratio 1.81, 95% CI 1.61–2.01, for a 10-fold increase in DCCD + 1). Even patients with a positive DCCD ≤3 had an increased risk of dying from melanoma compared to patients with DCCD = 0 (*p = *0.04; hazard ratio 1.63, 95% CI 1.02–2.58). Upon multivariable testing DCCD was a stronger predictor of death than histopathology. The final model included thickness, DCCD, and ulceration (all *p<*0.001) as the most relevant prognostic factors, was internally validated by bootstrapping, and provided superior survival prediction compared to the current American Joint Committee on Cancer staging categories.

**Conclusions:**

Cancer cell dissemination to the sentinel node is a quantitative risk factor for melanoma death. A model based on the combined quantitative effects of DCCD, tumor thickness, and ulceration predicted outcome best, particularly at longer follow-up. If these results are validated in an independent study, establishing quantitative immunocytology in histopathological laboratories may be useful clinically.

*Please see later in the article for the Editors' Summary*

## Abstract 2

In this study, Klein and colleagues investigated the impact of minimal cancer sentinel lymph node spread and of increasing numbers of disseminated cancer cells on melanoma-specific survival. The authors found that cancer cell dissemination to the sentinel node is a quantitative risk factor for melanoma death and the best predictor of outcome was a model based on combined quantitative effects of DCCD, tumor thickness, and ulceration.

*Please see later in the article for the Editors' Summary*

## Editors' Summary

**Background:**

Because the skin contains many different cell types, there are many types of skin cancer. The most dangerous type—melanoma—develops when mutations occur in melanocytes, the cells that produce the pigment melanin. Less than 5% of skin cancers are melanomas, but melanoma causes most skin cancer deaths. Early signs of melanoma are a change in the appearance of a mole (a pigmented skin blemish) or the development of a new and unusual pigmented lesion. If these signs are noticed and the melanoma is diagnosed before it has spread from the skin into nearby lymph nodes and other tissues, surgery often provides a cure. For advanced melanomas, the outlook is generally poor, although novel therapies may prolong a patient's life.

**Why Was This Study Done?:**

When a person is diagnosed with melanoma, it is important to “stage” the tumor. Knowing the extent and severity of the melanoma helps oncologists plan treatments and estimate their patients' likely outcomes. The detection of isolated melanoma cells in sentinel lymph nodes (the nodes to which cancer cells are most likely to spread from a primary tumor) is included in melanoma staging recommendations. However, finding rare tumor cells in sentinel lymph node biopsies by examining the tissue requires the analysis of many slides from each node removed from the patient and is extremely time-consuming. In this study, the researchers investigate the predictive value of a quantitative immunocytological assay that involves disaggregation of the sentinel node and detection of disseminated cancer cells (DCCs) by immunostaining for gp100 (a marker for melanoma cells). They also use this new assay to examine the effect of increasing numbers of DCCs on melanoma-specific survival.

**What Did the Researchers Do and Find?:**

The researchers used routine histopathology and immunocytology to analyze 1,834 sentinel lymph nodes from 1,027 patients with melanoma who underwent sentinel lymph node biopsy at one German hospital. For immunocytology, the researchers recorded the number of gp100-positive cells per million lymph node cells (the DCC density). During follow-up, 138 patients (13.4%) died from melanoma. The results indicated that increased DCC density was associated with an increased risk of death due to melanoma. Specifically, every 10-fold increase in DCC density + 1 was associated with a near doubling of the risk of death from melanoma (a hazard ratio of 1.81). Even patients with three or fewer gp100-positive cells per million lymph node cells had an increased risk of dying from melanoma compared to patients with no gp100-positive cells (hazard ratio 1.63). When other predictors of outcome such as age and primary tumor location were taken into account, DCC density was a stronger predictor of death than histopathology. Finally, a survival model that included tumor thickness, tumor ulceration, and DCC density provided survival prediction superior to that of a model based on the current standard staging recommendations.

**What Do These Findings Mean?:**

These findings show that quantification of cancer cell dissemination from melanomas to sentinel lymph nodes is feasible and can be combined with other characteristics of the primary tumor to provide an accurate prediction of outcomes for individual patients with melanoma. Notably, the new prediction model identifies a group of patients at high risk of progression for whom the current clinical standard underestimates the risk of death. These patients may benefit from adjuvant therapies, so the new analysis presented in this study may help to stratify patients for clinical trials. Importantly, quantitative immunocytology and the new model, although internally validated in this study, need to be validated in an independent group of patients before they can be considered for routine clinical use. If external validation is successful, quantitative immunocytology, which is much less labor-intensive than histopathology, has the potential to change the routine clinical care of patients with melanoma and probably with other solid tumors, conclude the researchers.

**Additional Information:**

Please access these websites via the online version of this summary at http://dx.doi.org/10.1371/journal.pmed.1001604.

- The US National Cancer Institute provides information for patients and professionals on melanoma, cancer staging, and sentinel lymph node biopsy (in English and Spanish)
- The nonprofit organization American Cancer Society provides information in several languages on cancer and how it develops and specific information on melanoma, including the AJCC system for staging and personal stories
- The UK National Health Service Choices website includes an introduction to cancer and a page on melanoma that includes personal stories
- The nonprofit organization Cancer Research UK provides basic information about cancer and detailed information on melanoma

## Introduction

For melanoma staging, sentinel node biopsy has been established to assess melanoma cell dissemination and has become the most widely used procedure to determine the regional lymph node status in patients with cutaneous melanoma [1],[2]. Because an evidence-based lower threshold for clinically relevant melanoma spread could not be defined [3], the detection of isolated melanoma cells in sentinel nodes was included in the latest American Joint Committee on Cancer (AJCC) staging recommendations [4]. However, the prognostic value of small tumor deposits in sentinel nodes is not unanimously accepted [5]–[7]. One potential reason for this lack of confidence may be that, unlike measurements of primary tumor thickness, highly sensitive methods for precise and direct quantification of sentinel node involvement by histopathology, optimal sample preparation, and screening are still lacking. The chance of detecting rare tumor cells by histopathology depends on the number of sections screened [8], and extensive histopathological protocols [7],[9]–[11] can achieve a detection rate of 30% but require the analysis of 24 to 36 slides per node [7]. Since this translates into 42 h of examination time for about ten melanoma patients a week [12], it is obviously impracticable for many institutions. Because of a lack of standardization [7],[10],[11],[13]–[16], the accuracy of sentinel node analysis has been limited for the mentioned practical reasons.

We previously developed a quantitative immunocytological assay to identify early cancer spread in sentinel nodes [17]. In this assay, the sentinel node is disaggregated, and disseminated cancer cells (DCCs) are detected by immunostaining for gp100, an antigen involved in melanin synthesis, using the HMB45 antibody [17]. The number of DCCs per 10^6^ isolated cells defines the DCC density (DCCD). In the current study we applied this assay to a prospective cohort of 1,027 patients. The aim was to evaluate its predictive value as a quantitative variable in comparison to qualitative routine histopathology and to address the role of minimal tumor seeding in the survival of melanoma patients.

## Methods

### Ethics Statement

Our study complied with the guidelines of the Declaration of Helsinki. As such, the institutional review boards of the University of Tübingen (ethics vote number 5/99) and the University of Regensburg (ethics vote number 07/79) approved the study. All patients provided written informed consent to examination of their sentinel nodes by disaggregation immunocytology, to the recording of their follow-up data in the Central Malignant Melanoma Registry of the German Dermatological Society, and to the molecular characterization of the isolated cells. Control (non-melanoma) nodes were obtained from patients with chronic venous insufficiency in whom a lymph node was removed during crossectomy, or from non-melanoma skin cancer patients. Sample acquisition was in agreement with the rules of the Ethics Committee of the University of Tübingen for use of waste tissue. No personal data were recorded from the control patients.

### Patients

From February 8, 2000, to June 16, 2008, we enrolled 1,154 patients who underwent lymphatic mapping and sentinel node biopsy at the University Hospital Tübingen, Germany, for histopathologically proven first invasive primary cutaneous melanoma. At the Department of Dermatology, University Hospital Tübingen, sentinel node biopsy is generally recommended for patients with melanoma lesions with a Breslow's tumor thickness of ≥1.0 mm, with primary tumors of Clark level IV or V, or with tumors of any Breslow's thickness but showing regression or ulceration. Twelve patients without other risk factors requested sentinel node biopsy although their melanomas were thinner than 1 mm. The preoperative staging to exclude metastatic disease consisted of a physical examination, ultrasound examination of regional lymph nodes and the abdomen, chest X-ray, and computed tomography brain scans. For final analysis 119 patients were excluded as they had a follow-up of less than 3 mo, and eight patients were excluded because of missing information about primary tumor thickness. The remaining 1,027 patients (Table 1) included 322 whose DCCD results have been reported in an interim analysis [17], however without follow-up information. Skin draining control lymph nodes (*n = *58) were obtained from 52 non-melanoma patients (52 skin draining nodes from nonmalignant conditions, six sentinel nodes from non-melanoma skin cancer patients) and disaggregated, stained, and evaluated identically to the lymph nodes of melanoma patients.

**Table 1:** Baseline characteristics of study cohort.

Characteristic	Number of Patients	Percentage	Median	Range	Interquartile Range
**Gender**					
Female	449	43.7			
Male	578	56.3			
**Age (years)**			58	10–86	44–69
**Breslow's thickness (mm)**			1.85	0.24–20.00	1.25–3.00
**Ulceration**					
No	738	71.9			
Yes	289	28.1			
**Localization**					
Extremities	500	48.7			
Trunk or head	527	51.3			
**Nodal status histopathology**					
Negative	888	86.5			
Positive	139	13.5			
**Clinical stage** a					
IA	76	7.4			
IB	393	38.3			
IIA	234	22.8			
IIB	138	13.4			
IIC	47	4.6			
IIIA	73	7.1			
IIIB	66	6.4			
**DCCD**			0.5	0–950,000	0–4

a Clinical stage according to the AJCC 2009 classification [4]. For nodal status only results of histopathology were taken into account. Information about mitotic rate was not available.

### Lymphatic Mapping, Sentinel Node Biopsy, and Tumor Cell Detection

Cutaneous lymphoscintigraphy, sentinel node biopsy, and sample preparation were performed as previously described [17] with minor modifications. From the beginning of the study until 31 July 2003, the procedure was as follows. The lymph node was cut along its longitudinal axis for histopathological and immunocytological examination. One half of the sentinel node was fixed in 3.5% formaldehyde, paraffin-embedded, and subjected to standard histopathological treatment, which included hematoxylin and eosin staining and immunohistochemistry on three 4-µm paraffin sections from the central level. From 1 August 2003 until the end of the study, the procedure was as follows. The lymph nodes were cut perpendicularly to the long axis [15]. For histopathology, 2-mm slices were cut after formalin fixation of the tissue. Hematoxylin and eosin staining and immunostaining (using antibodies directed against S100, HMB45, and Melan-A) of sections from each level was performed as described above. Thus, the total number of sections examined per node varied according to the size of the node. However, in all cases at least two levels (with four 4-µm sections each) were examined. The complete histopathological workup of the lymph nodes was done at the Department of Pathology, University of Tübingen, without knowledge of the immunocytological gp100 result. A patient was documented as histopathologically positive if at least one node was considered positive by the histopathological examination. The patients with isolated tumor cells were considered histopathologically positive.

Quantitative immunocytology was performed immediately after sentinel node biopsy at the Department of Dermatology, University of Tübingen, using the other unfixed half of the lymph node [17]. The lymphatic tissue was cut into 1-mm pieces and disaggregated mechanically into a single-cell suspension by rotating knifes (DAKO Medimachine, DAKO), washed with HBSS (Life Technologies), and centrifuged on a density gradient made of a 60% Percoll solution (Amersham). Cells were counted using a Neubauer counting chamber. Per slide, 10^6^ cells from the interphase were then dispensed onto adhesion slides (Menzel) in a volume of 1 ml of PBS. After sedimentation for 1 h, the slides were air-dried overnight. Immunocytological staining was carried out with the alkaline phosphatase/anti-alkaline phosphatase method using primary antibodies against gp100 (HMB45, DAKO) and Melan-A (A103, DAKO), and 5-bromo-4-chloro-3-indolyl phosphate/NBT (DAKO) as substrate, yielding a blue reaction product. A lymph node was defined as gp100 positive or Melan-A positive if it contained at least one gp100-positive or one Melan-A-positive cell, respectively. The number of positive cells per million isolated cells was recorded after screening of the slides by a technical assistant and final evaluation by a dermatologist, both experienced in evaluation of cytological preparations. The recording was done without knowledge of the histopathological findings or other clinical data. Positive preparations were air-dried or stored for a maximum of 4 d in PBS until cell isolation for genomic analyses.

### gp100/Melan-A Double Staining

For double immunofluorescence staining, additional slides were stained with primary antibodies against MART-1/Melan-A (rabbit monoclonal IgG, Epitomics) and gp100 (clone HMB45, mouse monoclonal IgG, DAKO). The cells were visualized after staining with Alexa Fluor 555 (donkey anti-rabbit IgG, Invitrogen) and Alexa Fluor 488 (donkey anti-mouse IgG, Invitrogen) and counterstained with 4′,6-diamidino-2-phenylindole (DAPI).

### Single-Cell Comparative Genomic Hybridization

Single-cell comparative genomic hybridization (CGH) was performed as previously described [18],[19]. In brief, proteinase K was used to digest cellular proteins after isolation, the single-cell genome was digested using MseI, adaptors were ligated to the 5′ overhangs, and the DNA fragments were amplified by PCR, resulting in an MseI representation of a single-cell genome. The reagents and protocol are now commercially available as kit (Ampli1, Silicon Biosystems). These amplicons were labeled and hybridized onto metaphase spreads or an Agilent 180 K microarray for array CGH [35]. Histograms for the CGH data were generated using the online algorithms at http://progenetix.net [20]. Twelve of the 46 patients from whom we isolated gp100-positive cells for the had samples with a median DCCD of 2 gp100-positive cells per million isolated cells (range 1 to 7) and were included solely to investigate the genomes of the early DCCs.

### Statistical Analyses

Melanoma-specific survival rates were calculated from the date of sentinel node biopsy until death from melanoma or the last follow-up. The 5-y survival percentages are derived from the Kaplan-Meier survival estimates, F(*t*). The 95% confidence intervals were based on the log(−log F(*t*)) transformation as described by Kalbfleisch and Prentice [21]. We calculated Pearson's correlation coefficient of log(DCCD + 1) and log(Melan-A + 1) for assessing the association of DCCD and Melan-A. For the comparison of positive DCCD values among subgroups of the other six prognostic variables we used either two-sample *t*-tests or one-way ANOVA after logarithmic transformation, i.e. we compared geometric means.

We used dot plots together with quartiles to show the differences in the distribution of DCCD values among the groups defined by the other variables.

We used univariable Cox regression models for the following seven predictors: gender, age, Breslow's thickness, ulceration, localization, nodal status pathology, and DCCD. A log transformation was used for the variable tumor thickness, and the logarithm of DCCD + 1 was used for the variable DCCD; hazard ratios are reported together with their 95% confidence intervals. *p*-Values are given for the likelihood ratio tests of the Cox models.

In addition, we calculated hazard ratios after grouping of the three continuous variables age, thickness, and DCCD. We plotted the hazard ratios of the models with the continuous and the grouped data for DCCD and tumor thickness in order to verify a linear model on a logarithmic scale.

For multivariable Cox regression analyses we adopted the model selection criterion according to Schwarz [22] and used the minimal value of the Bayes information criterion (BIC) to select the optimal model. This quantity is the sum of twice the negative log likelihood of the model plus the number of parameters times the logarithm of the sample size. Instead of evaluating the likelihood for all 128 ( = 2^7^) possible models we started with the model that included all seven variables, and then successively deleted the variable with the highest *p*-value. This approach finds the model with the lowest BIC value for a given number of variables, which was verified by calculating the BIC value for all 128 models.

For internal validation we used 100 bootstrap samples and calculated Harrell's *c*-index with and without correction for optimism [23]. We calculated Harrell's *c*-index instead of Somer's *D* because Harrell's *c*-index (*c* = (*D*+1)/2) estimates the proportion of concordant pairs among all comparable pairs of patients. We proceeded as follows. Step 1: we determined *c*_app_ from our model as selected using the BIC criterion. Step 2: we generated 100 bootstrap samples from the original dataset by sampling with replacement. Step 3: for each of these 100 bootstrap samples the same model selection procedure as for the original dataset was applied. Step 4: for each of the 100 bootstrap samples we calculated the *c*-index *c*_boot_. Step 5: the 14 different models found in step 3 were applied to the original dataset, and the corresponding *c*-indices *c*_orig_ determined. Step 6: the average optimism of the fit was calculated as *c*_orig_−*c*_boot_. Step 7: the bootstrap-corrected performance of the original stepwise model was calculated as *c*_app_−(*c*_orig_−*c*_boot_).

In order to verify the proportional hazards assumption of the Cox model we divided the patients into two groups for each of the three final predictors and plotted the ratio of their cumulative hazard functions as a function of time. According to the Cox model this ratio should stay constant. As an alternative to the Cox model we used the lognormal distribution as a model for predicting outcome by DCCD, thickness, and ulceration. The lognormal model allows determination of the maximum time-dependent hazard rate and the time at which it occurs (formulas for the lognormal survival probability are in Text S1). We used the lognormal model to determine the 5-y survival probabilities by a nomogram [24].

### Model Validation and Comparison

To assess the goodness of fit of the models (Table S3), we divided the 1,027 patients into 18 subgroups according to the following criteria: three groups of DCCD values, with DCCD = 0 assigned the value 0, 0< DCCD <100 assigned the value 1, and DCCD ≥100 assigned the value 2; three groups of tumor thickness, with tumor thickness ≤2 mm assigned the value 1, 2 mm < tumor thickness ≤4 mm assigned the value 2, and tumor thickness >4 mm assigned the value 3; and ulceration no/yes. We restricted the analysis to 18 subgroups because otherwise the number of patients per subgroup would be too small. Only a slight difference in the hazard ratio was observed between the categories 0< DCCD ≤3 and 3< DCCD <100, as shown in Table 2. These categories were therefore combined. For each of the 1,027 patients, we calculated the expected failure probability for the individual follow-up times. The observed numbers of deaths in the 18 subgroups were compared to the expected numbers of deaths by the chi-square statistics. The goodness of fit of the models was compared by the sum of the 18 chi-square values, taking into account the number of degrees of freedom, which depends on the number of estimated parameters. Since the models were not nested we did not perform likelihood ratio tests. We provide the chi-square statistics only for descriptive purposes. The grouping of patients according to AJCC criteria was based on the AJCC 2009 recommendations (which include assignment of isolated tumor cells as nodal positive) [4], with the exception that mitotic rate could not be included because it had not been assessed at the beginning of the study. For the AJCC grouping, the nodal status was determined by histopathology and not by immunocytology.

**Table 2:** Univariable survival analyses.

Variable	Number of Patients	Median (Range)	Hazard Ratio^a^	95% CI	*p*-Value
**Gender**					
Female	449		1		
Male	578		1.43	0.95–1.89	0.10
**Age**		58 (10–86) y	1.02	1.01–1.04	<0.001
<58 y	508		1		
≥58 y	519		1.79	1.28–2.54	<0.001
**Breslow's thickness**		1.85 (0.24–20.00) mm	18.63	10.68–32.24	<0.001
≤2.00 mm	579	1.30 mm	1		
2.01–4.00 mm	315	2.75 mm	2.89	1.91–4.42	<0.001
4.01–8.00 mm	106	5.00 mm	6.78	4.26–10.76	<0.001
≥8 mm	27	10.00 mm	11.66	5.81–21.73	<0.001
**Ulceration**					
No	738		1		
Yes	289		3.79	2.71–5.32	<0.001
**Localization**					
Extremities	500		1		
Trunk or head	527		1.43	1.02–2.02	0.04
**Nodal status pathology**					
Negative	888		1		
Positive	139		4.34	3.04–6.11	<0.001
**DCCD**		0.5 (0–950.000)	1.81	1.61–2.01	<0.001
DCCD = 0	502	0	1		
0< DCCD ≤3	249	1	1.63	1.02–2.58	0.041
3< DCCD <100	194	9	1.66	1.01–2.68	0.048
100≤ DCCD	82	1,081	8.04	5.13–12.60	<0.001

a In addition to the hazard ratios for the groups we also provide unit hazard ratios for the continuous variables age, tumor thickness, and DCCD, together with their *p-*values. Tumor thickness and DCCD + 1 enter the Cox model with their common logarithms; thus, a unit corresponds to a 10-fold increase. For thickness, a unit corresponds to an increase from 1.3 to 13 mm. For DCCD a unit corresponds to an increase from zero to nine cells, because we add one to all cell counts before taking logarithms.

For the parametric model with the variables thickness, DCCD, and ulceration, each individual patient was characterized by his or her risk score. This score was a linear combination of the logarithms of tumor thickness and DCCD and of ulceration. The purpose was to assess the goodness of fit of our model in four well-defined groups of patients. Since the precision of Kaplan-Meier estimates depends essentially on the number of deaths in a sample, we wanted to achieve similar precision in all four groups. To this end the 138 patients who died from melanoma were divided into four groups with increasing risk scores of death, as defined by the survival model. Subsequently, the risk thresholds of the four groups were applied to all the patients. For given values of the three variables included in the predictive models each patient can uniquely be assigned to one of the four groups. All four groups differed from each other significantly (all *p-*values <0.001 in the log-rank test comparing group 2 to group 1, group 3 to group 2, and group 4 to group 3).

We next compared the goodness of fit of the parametric model with the variables thickness, DCCD, and ulceration with a Cox model using the same variables. We found that the parametric model performed better (*p = *0.13, sum of χ^2^ values = 21.1) than the corresponding Cox proportional hazards model (*p = *0.03, sum of χ^2^ values = 27.4) after grouping all 1,027 patients into the 18 risk groups (see above and Table S3).

To assess whether a model that included the information on Melan-A staining in addition to gp100 staining could further improve outcome prediction we compared our model based on DCCD (the maximum number of gp100-positive cells per million isolated cells per patient), thickness, and ulceration with the model DCCD 2 (defined as the maximum number of gp100- or Melan-A-positive cells per million isolated cells), thickness, and ulceration.

We found that the model that included the information on Melan-A staining was not superior to gp100-based DCCD reporting (*p = *0.09, sum of χ^2^ values = 22.7; Table S3). Finally, we compared the predictions based on our parametric model and on AJCC staging [4]. The goodness of fit for our model (*p = *0.13, sum of χ^2^ values = 21.1) was much better than that of the AJCC staging model (*p<*0.0002, sum of χ^2^ values = 36.7). For details and data, see the Table S3.

For comparison with the AJCC model, the patients were originally divided into three groups according to the differences in survival predictions between the two models. For individuals in Group S1 of Table S2, the survival probability for the new model was at all times greater than the survival probability according to the AJCC model; the absolute percentage difference was greater than 13%. In Group S2 the survival probability for the new model was at all times smaller than the survival probability according to the AJCC model; the maximum absolute percentage difference was greater than 13%. For Groups S1 and S2, the maximum absolute percentage difference of 13% was chosen because it exceeds the maximum absolute percentage differences for those patients for whom the survival in one model was not always greater than the survival in the other model. The remaining patients formed Group S3.

The risk scores were the sums of the products of the individual predictors multiplied by the corresponding regression coefficients. Since some tumor thicknesses were less than 1 mm, we also obtained negative risk scores because tumor thickness was on a log scale.

We performed statistical analyses with JMP (version 10.0.2).

## Results

### Patients

The final analysis included a total of 1,834 sentinel nodes from 1,027 patients examined by quantitative immunocytology and by histopathology. The baseline characteristics of all 1,027 patients are summarized in Table 1. The median follow-up was 49 mo (range 3 to 123 mo), with 370 (36%) patients having a follow-up of at least 5 y. During follow-up, 138 of 1,027 patients (13.4%) died from melanoma. The 5-y melanoma-specific survival probability for all patients was 86% (95% CI 83%–88%).

### Detection of Disseminated Melanoma Cells by Immunocytology

**Figure 1:**
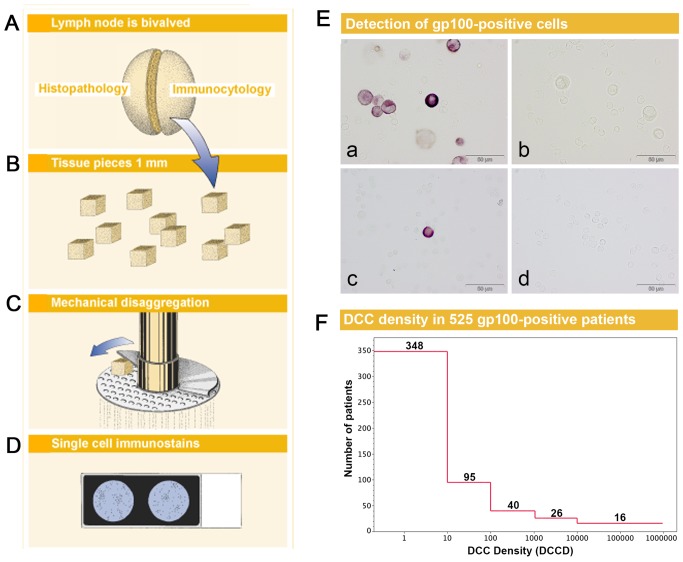
Sample preparation, melanoma cell detection, and distribution of disseminated cancer cell densities. (A) The sentinel nodes were split into halves for routine histopathology and immunocytology. (B–D) After mincing (B), the small pieces were subjected to mechanical disaggregation (C), and single-cell suspensions were added to adhesion slides (D). (E) The detection of gp100-positive cells among the unstained lymphocytes. (a) gp100 staining of Mel Ho cell line cells mixed among peripheral blood leukocytes used as positive control. (b) Isotype control of positive control. Note that the large melanoma cells can be identified by size among the peripheral blood leukocytes and are completely unstained. (c) gp100-positive cell from melanoma patient with DCCD = 1. (d) Isotype control of the melanoma patient in (c). All four samples are taken from the same experiment. (F) The distribution of DCCD (number of DCCs per 10^6^ isolated cells) in the sentinel nodes of 525 gp100-positive patients. Note that some patients had DCCD <1. The 502 zero values for DCCD are not included.

The underlying rationale of our detection assay (Figure 1A–1E) comprises two aspects. First, the spatially inhomogeneous distribution of melanoma cells may be equalized by lymph node disaggregation and generation of a single-cell suspension, which facilitates melanoma cell detection when only parts of the sample are screened; second, the number of melanoma cells can be counted and the amount of analyzed tissue can be quantified by referencing the number of melanoma cells to a defined number of isolated lymph node cells. This allows determining the DCCD, i.e. the number of DCCs per 1 million isolated cells.

We first assessed whether gp100-positive cells could be detected in skin draining nodes from non-melanoma patients. These lymph nodes were prepared identically to the sentinel nodes from melanoma patients (Figure 1B–1D), except that lymph nodes from cancer patients had to be split in half to provide tissue for routine histopathology. We could not detect a single gp100-positive cell among 171×10^6^ cells isolated from 58 non-melanoma skin draining lymph nodes.

**Figure 2:**
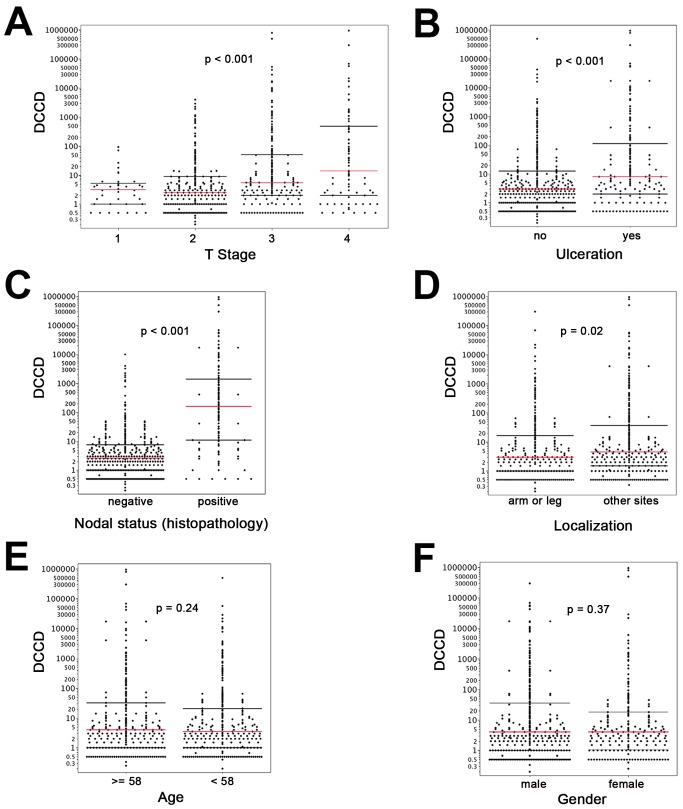
DCCD and standard clinical prognostic factors. For all panels, the red lines indicate the medians, and the black lines the 25th and 75th percentiles. (A) All DCCD values are plotted according to the T stage of the primary melanoma. (B) DCCD and ulceration state of the primary melanoma. (C) DCCD and status according histopathological routine analysis of the other lymph node half. (D) DCCD and localization of the primary melanoma. (E) DCCD and age category (<58 y and ≥58 y) of patient. (F) DCCD and gender of patient.

On the other hand, we detected gp100-positive cells in the lymph nodes of 525 of the 1,027 melanoma patients (51%). Whenever enough cells were isolated from the lymph node half for immunocytology, we aimed to screen 2×10^6^ lymph node cells, i.e., two slides, per patient. The median number of slides screened per node was two (range 10^4^ to 6×10^6^ cells). The median DCCD in patients with DCCD >0 was 4 gp100-positive cells per million isolated cells (ranging from 0.2 to 950000; Figure 1F). We evaluated the relation of DCCD with the six established prognostic factors (Figure 2). Geometric mean values of DCCD were significantly higher in thicker and ulcerated melanomas (*p<*0.001), in melanomas located at other sites than the extremities (*p = *0.02), and in patients with a pathologically positive sentinel node (*p<*0.001; Figure 2).

However, severe concerns about the immunocytological assay may be raised by (1) the loss of architectural information, which helps to differentiate between intra-lymphatic nevi and colonies of melanoma cells, (2) the difficulty to identify melanoma colonies by morphological criteria, and (3) the fact that the gp100 antigen for melanoma detection may be down-regulated. We addressed these concerns by careful evaluation of lymph node preparations from melanoma and non-melanoma patients using a second melanoma-associated antibody directed against Melan-A and by genetic analysis of the gp100-positive cells.

We found Melan-A-expressing cells in three out of 38 (8%) control lymph nodes, all of which were gp100 negative. We then proceeded to determine the detection rate of the two antibodies for melanoma cells in sentinel nodes from melanoma patients with histopathologically proven lymphatic spread by applying a double staining method. Because Melan-A-positive cells were detected in 8% of control nodes, we restricted the direct comparison of gp100- and Melan-A-positive cells to samples from histopathologically positive nodes, where expression of Melan-A by benign cells would be less likely to confound the analysis. Evaluating 3,055 cells from 43 nodes of 41 patients after immunofluorescence double staining, we found that 39 of 43 (91%) lymph nodes harbored cells positive for gp100 and Melan-A (Figure S1), one of 43 (2%) had only gp100-positive cells, and three of 43 (7%) had only Melan-A-positive cells.

These data suggest that the slightly higher detection rate of the Melan-A antibody does not outweigh the lower specificity as determined by the control samples. However, to completely rule out that gp100-negative DCCs comprise a relevant confounding factor, additional slides were stained using the Melan-A antibody in 710 patients. Comparing the gp100 and Melan-A staining results of these 710 patients, we confirmed the high correlation of gp100 and Melan-A staining (*r* = 0.83, *p*<0.001) that we had previously seen by double immunofluorescence. As detailed in Table S3, we found that the inclusion of Melan-A did not improve the prognostic power of the gp100-based immunocytological assay.

### Genetic Characteristics of Disseminated Melanoma Cells

**Figure 3:**
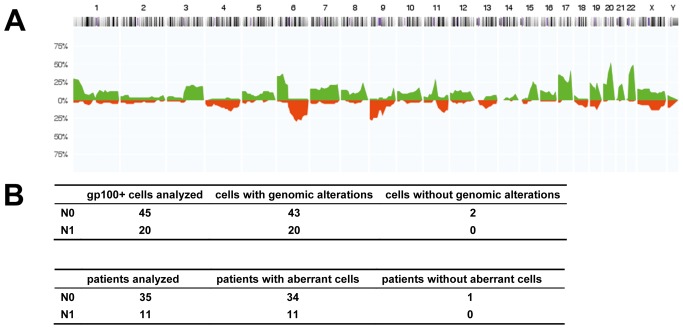
Chromosomal aberrations of isolated gp100-positive cells. (A) CGH profiles of gp100-positive cells (*n = *57 cells, all displaying chromosomal aberrations in metaphase CGH). The histogram displays the percentage of cells with specific chromosomal alterations. The chromosomal position is indicated by the horizontal chromosome ideogram (from Chromosome 1 on the left to the X and Y chromosomes on the right). Copy number gains for each region are depicted in green, and copy number losses are depicted in red. (B) Summary of findings for all 65 cells and 46 patients with respect to the lymph node status as determined by routine histopathology (N0 = histopathologically negative nodes, N1 = histopathologically positive nodes). After analysis of the eight cells with normal karyotypes in metaphase CGH by array CGH, all but two cells displayed genomic aberrations. One of these two cells was isolated from a sample where an additional gp100-positive cell displayed alterations, while the other cell was the only gp100-positive cell detected in that patient.

Since all these findings provided *indirect* support that gp100-positive cells represent DCCs, we searched for *direct* evidence of their malignant origin. We randomly isolated 65 gp100-positive cells from 46 patients for a whole-genome screen of chromosomal aberrations by CGH. The DCCD values of these patients ranged from 0.2 to 800,000 gp100-positive cells per million isolated cells (median = 8), and we analyzed between one and three cells per patient. Metaphase CGH provided direct proof for the malignant origin of 57 gp100-positive cells (Figure 3A), while eight cells displayed normal karyotypes. As metaphase CGH has a resolution of 10–20 Mb, we subsequently applied array CGH [35], which has a resolution of <1 Mb, to these eight cells. While we could not detect any aberration in two cells, the remaining displayed between one and ten changes (median = 4.5) ranging from 0.1 to 19 Mb (median = 2 Mb). In summary, 63 of 65 gp100-positive cells (97%) displayed genomic aberrations, which classified 45 of 46 patients (98%) as harboring cancer cells in their sentinel nodes. There was no difference for cells isolated from lymph nodes classified as negative or positive by routine histopathology, demonstrating that our assay is suited to correctly identifying melanoma cells without morphological assessment of tissue architecture (Figure 3B).

### Disseminated Cancer Cell Density and Melanoma-Specific Survival

**Figure 4:**
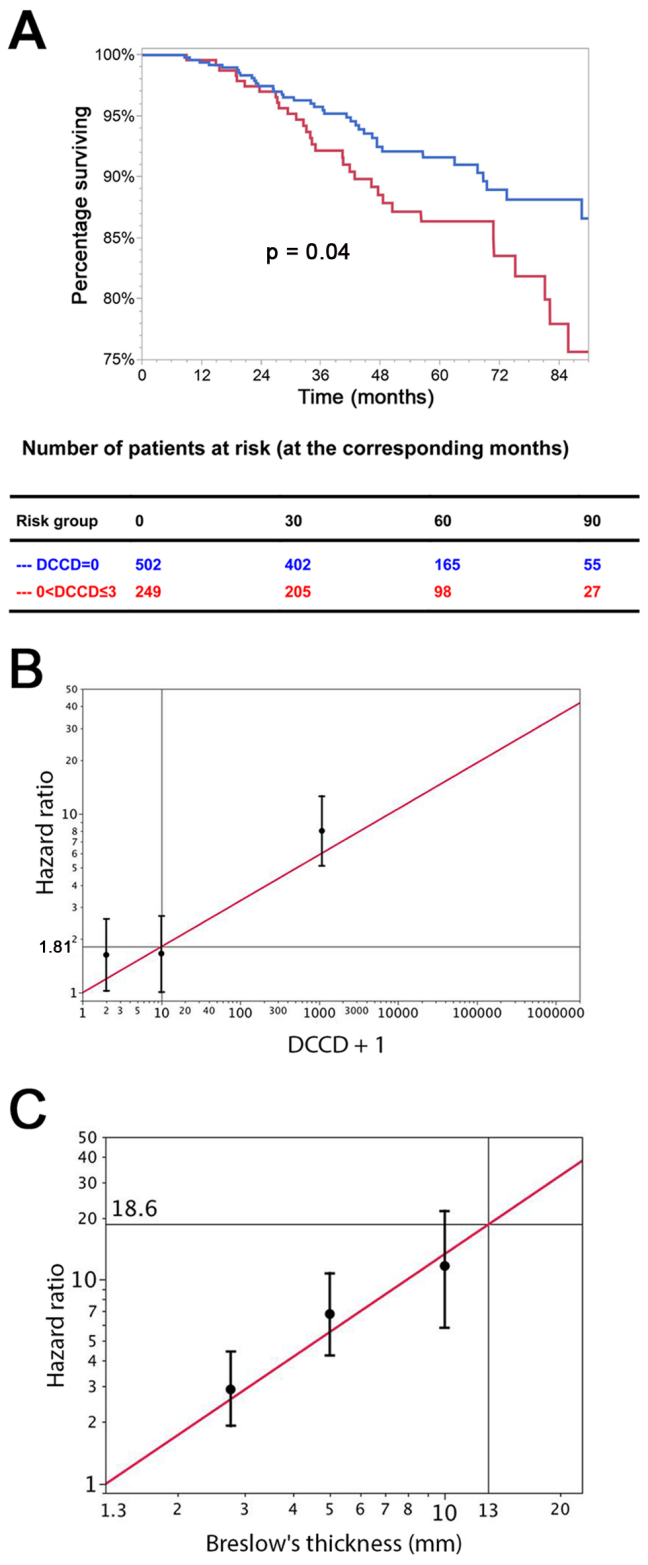
The prognostic impact of disseminated cancer cells in sentinel nodes. (A) The figure shows the Kaplan-Meier survival estimates for 502 patients with DCCD = 0 (blue line) and for 249 patients with 0< DCCD ≤3 (red line). Even patients with low numbers of DCCs had a significantly reduced survival in comparison to patients without DCCs (86% 5-y survival versus 92% 5-y survival; hazard ratio 1.63, 95% CI: 1.02–2.58). (B) Hazard ratios (in black) for the three DCCD categories of Table 2 together with their 95% confidence intervals as a function of their median DCCD values on a log scale. The red line provides the hazard ratios for the linear model where log(DCCD + 1) is entered as a continuous variable. The predicted curve is within the confidence intervals. The unit risk ratio 1.81 is the hazard ratio corresponding to a 10-fold increase of DCCD + 1. (C) Relation of Breslow's thickness and risk of death from melanoma. Hazard ratios (in black) are given for the three groups of Table 2 (melanomas with 2 mm ≤ tumor thickness ≤4 mm; 4< tumor thickness ≤8 mm; tumor thickness >8 mm) together with their 95% confidence intervals as a function of their median thickness values on a log scale. The red line shows the predicted hazard ratios for the model where log(thickness) enters as a continuous variable. We show only the values ≥1.3 mm, i.e., the median value of the reference group in Table 2. The predicted line is within the confidence intervals. The unit hazard ratio 18.6 corresponds to a 10-fold increase of thickness.

We evaluated DCCD as biomarker according to the REMARK criteria [25]. Of the standard prognostic factors, sentinel node histopathology (*p<*0.001), age (*p<*0.001), thickness (*p<*0.001), ulceration (*p<*0.001), and localization of the primary melanoma (*p = *0.04) were associated with poor outcome in the univariable Cox regression analyses (see Table 2 and Figure S2 for Kaplan-Meier estimates). Increasing DCCD values were negatively associated with the time to death from melanoma in the univariable Cox regression analyses (*p<*0.001). We assessed the prognostic impact of DCCD after categorizing the values into four groups (Table 2). We found that even the detection of low DCCD values (0<DCCD ≤3) conferred a significant risk of death (hazard ratio 1.63, 95% CI 1.02–2.58, *p = *0.04; Table 2 and Figure 4A) compared to patients without DCCs. Increasing hazard ratios were obtained for categories with higher DCCD values (Table 2). The relationship of increasing DCCD values and the hazard ratio is plotted in Figure 4B on the logarithmic scale. The unit risk ratio (corresponding to a 10-fold increase of DCCD + 1, e.g., from a DCCD of zero to a DCCD of nine) was 1.81 (95% CI 1.61–2.01), and a linear relation (on log scale) between DCCD and hazard ratio was identified (Figure 4B). A similar log-linear relationship was seen between tumor thickness and hazard ratio (Figure 4C).

We next performed stepwise multivariable Cox regression analysis starting with all six standard prognostic factors in addition to DCCD. After each step of the multivariable analysis the variable with the highest *p-*value was deleted (Tables 3 and 4). To identify the optimal model, we determined the BIC, which has a minimal value for the best model [22]. As can be seen from Table 4, the BIC value is lowest for the combined variables tumor thickness, DCCD, and ulceration, for which all *p-*values were below 0.001. The unit hazard ratios for this model were 6.96 (95% CI 3.61–13.28) for thickness, 1.43 (95% CI 1.27–1.61) for DCCD, and 2.04 (1.4–2.97) for ulceration. It should be noted that nodal status determined by routine histopathology had a maximum hazard ratio of 1.75 (95% CI 1.04–2.86) in multivariable analyses and was rejected already in step 3 (Table 3).

**Table 3:** Multivariable survival analyses: hazard ratios together with their 95% confidence intervals.

Step	Number of Predictors in Model	Breslow's Thickness^a^	DCCD^b^	Ulceration^c^	Age^d^	Nodal Status^e^	Localization^f^	Gender^g^
1	7	6.51 (3.32–12.58)	1.25 (1.06–1.46)	1.89 (1.30–2.76)	1.02 (1.01–1.03)	1.74 (1.03–2.85)	1.32 (0.91–1.93)	1.05 (0.73–1.52)
2	6	6.46 (3.31–12.45)	1.24 (1.06–1.46)	1.89 (1.30–2.76)	1.02 (1.01–1.03)	1.75 (1.04–2.86)	1.34 (0.94–1.92)	—
3	5	6.09 (3.11–11.77)	1.29 (1.11–1.50)	1.90 (1.31–2.78)	1.02 (1.00–1.03)	1.67 (1.01–2.72)	—	—
4	4	6.63 (3.42–12.71)	1.43 (1.27–1.60)	1.94 (1.33–2.84)	1.01 (1.00–1.03)	—	—	—
5	3	6.96 (3.61–13.28)	1.43 (1.27–1.61)	2.04 (1.40–2.97)	—	—	—	—
6	2	10.75 (5.86–19.54)	1.47 (1.31–1.65)	—	—	—	—	—
7	1	18.63 (10.68–32.24)	—	—	—	—	—	—

a log_10_ of tumor thickness;, hazard ratios for a 10-fold increase.

b log(DCCD + 1); hazard ratios for a 10-fold increase.

c Hazard ratios for ulceration versus no ulceration.

d Hazard ratios for one additional year.

e Hazard ratios for positive versus negative by routine histopathology.

f Hazard ratios for trunk or head versus extremities.

g Hazard ratio for male versus female.

**Table 4:** Multivariable survival analyses: model selection according to *p-*values and Bayes Information Criterion.

Step	Number of Predictors in Model	Breslow's Thickness	DCCD	Ulceration	Age	Nodal Status^a^	Localization	Gender	BIC
1	7	<0.0001	0.0068	0.0009	0.0051	0.0371	0.1380	0.8052	1,657.8
2	6	<0.0001	0.0069	0.0008	0.0050	0.0340	0.1035	—	1,650.9
3	5	<0.0001	0.0011	0.0008	0.0054	0.0475	—	—	1,646.6
4	4	<0.0001	<0.0001	0.0005	0.0127	—	—	—	1,643.6
5	3	<0.0001	<0.0001	0.0002	—	—	—	—	1,642.9
6	2	<0.0001	<0.0001	—	—	—	—	—	1,649.8
7	1	<0.0001	—	—	—	—	—	—	1,679.1

*p-*Values are for testing whether a hazard ratio equals 1; low BIC identifies best model.

a As determined by routine histopathology.

### Individual Risk Prediction by Tumor Thickness, Disseminated Cancer Cell Density, and Ulceration

**Figure 5:**
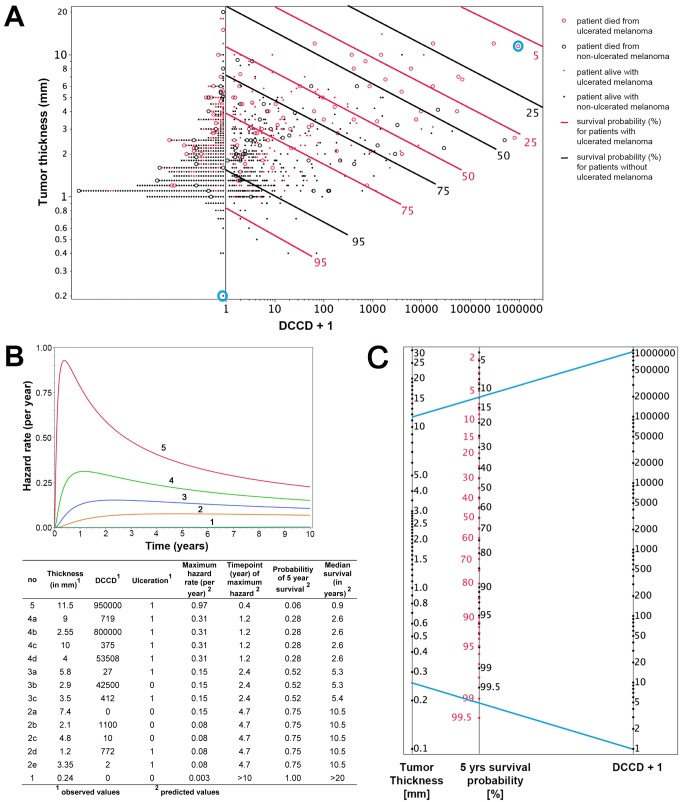
Individualized risk estimation over time. (A) The predicted 5-y survival by DCCD, tumor thickness, and ulceration for all 1,027 patients. Dots indicate patients who were alive at the end of follow-up; circles indicate patients who died from melanoma (*n* = 138). The red symbols indicate patients with ulcerated melanomas. The patients left of the vertical line at DCCD + 1 = 1 (*n* = 502) displayed no DCCs in their sentinel nodes. Dots located on the same continuous line represent patients with the same 5-y survival, which is depicted at the right end of the lines (red lines for the patients with ulcerated melanoma). For example, for Patient 1 and Patient 5 from (B) (circled in blue), the estimated 5-y survival of Patient 1 is >99.5%, while for Patient 5 it is about 6%. The parametric survival model is based on the lognormal distribution and includes DCCD, thickness, and ulceration. (B) Time-dependent hazard rates for 14 individual patients with five different hazard rates (1–5) with their observed thickness, DCCD, and ulceration status, and their corresponding predicted 5-y survival rates and median survival times. Different combinations of thickness, DCCD, and ulceration status may indicate the same risk of death from melanoma. In contrast to the Cox model, the predicted hazard ratios vary over time. (C) A nomogram enables estimating the 5-y survival probability. The 5-y survival probability is the value at the intersection of a straight line connecting DCCD and tumor thickness with the central straight line. For illustration, the blue lines exemplify the same Patients 1 and 5 that are marked in (A) and described in (B). Patient 5 had an ulcerated melanoma, and therefore the red scale must be used, while Patient 1 had a non-ulcerated melanoma, so survival probability is given on the black scale. Note that the nomogram has not been independently validated and therefore is not yet suitable for clinical use.

To fully exploit the power of our quantitative assay, we combined the three most important risk factors identified by multivariable analysis (tumor thickness, DCCD, and ulceration) for individual risk assessment at diagnosis and during follow-up. While results of Cox models represent a useful summary for the average hazard ratios, we observed that the assumption of proportional hazards was not fulfilled for tumor thickness and DCCD (Figure S3). Therefore, we employed a lognormal survival model based on tumor thickness, DCCD, and ulceration that allows the calculation of changes in individual risk over time and of the predicted 5-y survival for all 1,027 patients (Figure 5A). Figure 5A shows that patients with thin melanomas never harbored high DCC numbers in their lymph nodes and poorest outcome was seen for thick tumors and high DCCD. It should be noted that DCCD and tumor thickness are plotted on a logarithmic scale, and therefore the curves of equal 5-y survival probability appear as straight lines. On a linear scale these curves (isoboles) are convex (Figure S4), which indicates synergism [26]. Using this model, we calculated the time-dependent hazard rates for 14 individual patients with five different hazard rate curves (Figure 5B). This calculation revealed that DCC-negative and DCC-positive patients might display identical hazard rate functions (e.g., compare Patients 2a and 2b in Figure 5B) and also that the hazard rate peaks later in low-risk than in high-risk patients. Furthermore, tumor thickness, DCCD, and ulceration state can be integrated into a preliminary nomogram to determine the 5-y survival of individual patients (Figure 5C).

### Internal Validation of the Model

While the present survival model based on the predictors thickness, DCCD, and ulceration awaits validation by an independent multi-center study, we sought to validate it internally. For this we applied a bootstrapping approach [23]. We generated 100 bootstrap samples from the original dataset by sampling with replacement. For each of these 100 bootstrap samples the same model selection procedure as for the original dataset was applied. We obtained 14 different “best models” (Table 5). The present model was selected most often. The variable DCCD was included in 85 best models, whereas nodal status by routine histopathology was included in only 36 of the 100 models. Harrell's *c*-index, which estimates the probability of concordance between predicted and observed responses, for the present model was 0.763 in the original dataset. Harrell's *c*-index based on the current AJCC staging system was 0.737. This is significantly smaller (*p<*0.0001; McNemar's test). The bootstrap-corrected *c*-index [23] for the present model turned out to be 0.748, which is well above the value of 0.5 representing only random prediction ability.

**Table 5:** Variables included in the 14 best models found in the 100 bootstrap samples created for internal validation.

Model	Breslow's Thickness	DCCD	Ulceration	Age	Nodal Status^a^	Localization	Number of Bootstrap Samples with the Model
1	+	+	+	−	−	−	27
2	+	+	+	+	−	−	22
3	+	+	+	+	+	−	16
4	+	+	−	+	−	−	9
5	+	−	+	+	+	−	9
6	+	+	−	+	+	−	5
7	+	−	−	+	+	+	3
8	+	+	−	−	−	−	2
9	+	−	−	+	−	+	2
10	+	−	−	+	−	+	1
11	+	+	+	−	+	−	1
12	+	+	+	−	−	+	1
13	+	−	+	+	+	+	1
14	+	−	+	−	+	+	1
No of bootstrap samples with the variable included	100	85	78	68	36	9	

A plus sign indicates that the variable was included in the model; a minus sign indicates that it was not included.

a As determined by routine histopathology.

We then analyzed those patients for whom the predictions of the AJCC and the new model differed (survival probability in group S1: new model > AJCC; in group S2: new model < AJCC; group S3, remaining patients). We calculated the expected number of deaths at the observed follow-up time for each patient and compared this number with the observed number of deaths (Table S2). Only the new model provided an acceptable fit for all three groups. In Group S2 of Table S2 the number of deaths predicted in the AJCC model was significantly different from the number of observed deaths (*p*<0.0001).

Finally, we combined Groups S1 and S3 from Table S2 to form two groups. Group 1 now comprised patients for whom the novel model predicted better survival than the AJCC model and patients for whom the predictions of both models concurred. Patients for whom the novel model predicted a worse survival than the AJCC model formed Group 2.

**Figure 6:**
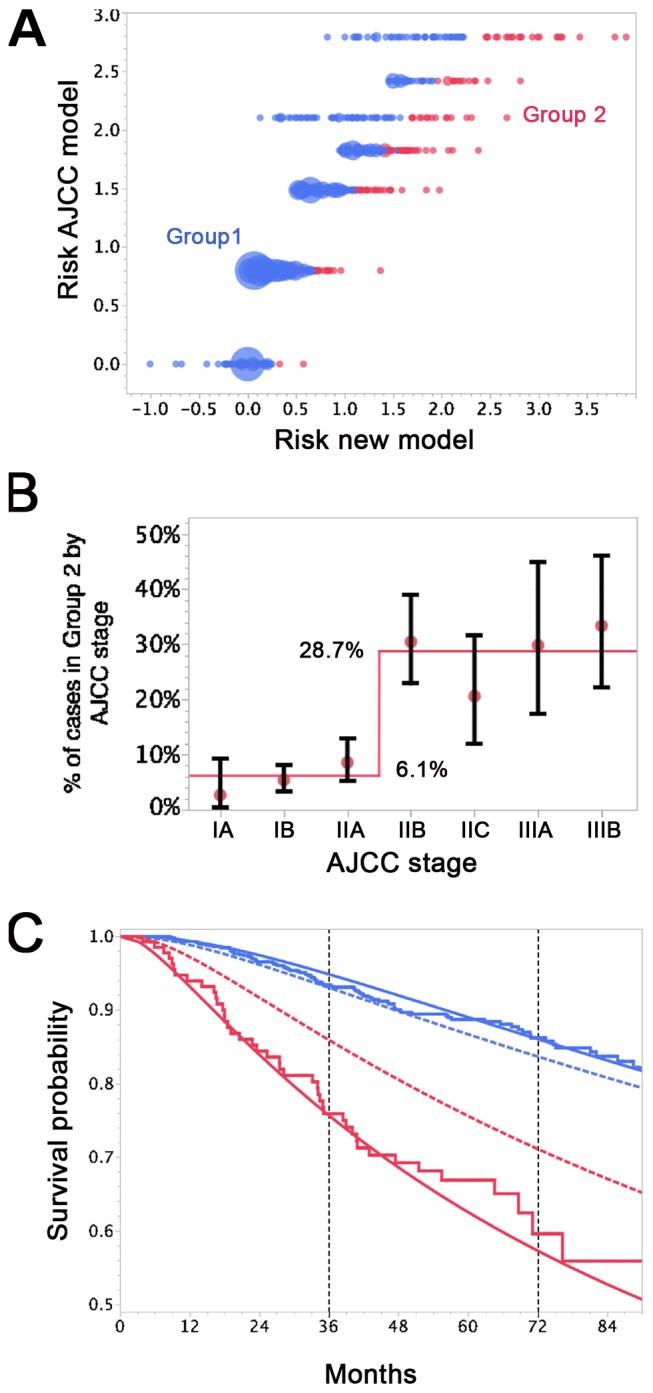
Differences in survival prediction between this study's model and the AJCC-based model. (A) Joint distribution of the individual risk scores for the 1,027 patients according to two predictive survival models: (1) “Risk new model” refers to the lognormal model with the predictors tumor thickness, DCCD, and ulceration; (2) “Risk AJCC model” refers to a lognormal model based on the seven categories of the AJCC classification (stage IA, IB, IIA, IIB, etc.). The risk scores are sums of products of the individual predictors multiplied with the corresponding regression coefficients. (Since some tumor thicknesses are less than 1 mm we obtain also negative risk scores because tumor thickness enters on a log scale.) The area of the bubbles is proportional to the number of patients with identical combinations of risk values. The largest bubble contains 30 patients. The smallest bubbles represent one patient each. In Group 2 (red; *n* = 136) the survival probability for a patient from the new model is at all times smaller than the survival probability according to the AJCC model. The remaining patients form Group 1 (blue; *n* = 891). Note that there are only seven risk groups for the AJCC model, while the new model exploits the full risk range. (B) Observed (red filled circles; together with the exact 95% confidence intervals) and fitted percentages of patients in Group 2. Almost 29% of patients with AJCC stage > IIA are in Group 2, whereas only about 6% of patients with AJCC stage < IIB are in Group 2. (C) Observed (Kaplan-Meier survival estimates; step functions) and predicted survival probabilities for the new model (continuous lines) and the AJCC model (dashed lines) for the Group 1 (blue) and Group 2 (red). Note the large difference between the AJCC model and the new model for Group 2 and the perfect fit of the observed survival and the new model's predictions for Group 1 after 60 mo.

Nearly 94% of AJCC low-risk patients (<IIB) were in Group 1 (Figure 6A and 6B). Nearly 29% of AJCC high-risk patients (>IIA) were in Group 2. Patients in Group 2 had higher DCCD values than patients in Group 1. The geometric means of DCCD + 1 are 2.23 (95% CI 1.98–2.51) in Group 1 and 80.53 (95% CI 59.54–108.94) in Group 2, respectively (*p*<0.0001; two-sample *t*-test for the logarithms).

For the time points 3 and 6 y after sentinel lymph node biopsy we determined which model provided a better fit for the survival of patients in Group 1 and 2. Kaplan-Meier plots demonstrated that the predicted and observed survival curves diverge particularly for the AJCC prediction of Group 2 patients (Figure 6C). For both time points 3 and 6 y (*p<*0.001 and *p<*0.01, respectively) the AJCC model significantly deviated from Kaplan-Meier estimates for Group 2 patients, whereas our model correctly predicted the number of deaths (Table 6).

**Table 6:** Kaplan-Meier estimates versus predicted deaths for a follow-up of 3 and 6 y by model and group.

Year of Follow-Up	Group	*n* (Percent)	Expected Deaths			*p*-Value	
			AJCC	New Model	Kaplan-Meier Estimates	AJCC Model^a^	New Model^a^
3	1	891 (86.8)	61.9	45.8	58.6	0.67	0.06
3	2	136 (13.2)	19.1	33.0	32.8	0.001^a^	0.97
6	1	891 (86.8)	145.1	124.0	123.0	0.07	0.93
6	2	136 (13.2)	39.2	58.0	55.0	0.01^a^	0.69

a Compared to Kaplan-Meier estimates.

For the low-risk patients of Group 1, both models provided acceptable fits, although we noted a borderline *p-*value (*p* = 0.06) for the new model at 3 y. However, the fit for predicted and observed survival becomes excellent for the new model over time (Figure 6C)—in line with the need for longer observation periods in low-risk patients. Thus, at 6 y follow-up there is perfect agreement for the new model, whereas the AJCC model overestimates the number of deaths (Figure 6C and Table 6).

**Figure 7:**
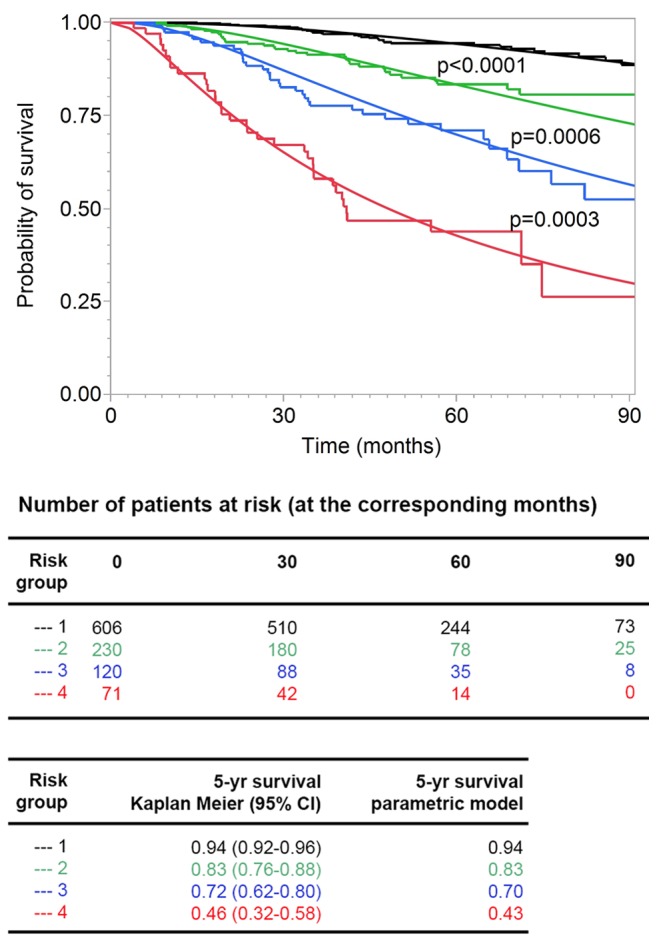
Goodness of fit of observed and predicted survival for four risk groups. The 138 patients who died from melanoma were divided into four groups (see Methods) with increasing risk of death, as defined by the survival model. Subsequently, the risk thresholds of the four groups were applied to all the patients. The Kaplan-Meier estimates (step functions) and the predicted survival curves (smooth curves) from the survival model are depicted for these four groups. All four of the groups differed from each other significantly (all *p-*values<0.001 in the log-rank test comparing group 2 to group 1, group 3 to group 2, and group 4 to group 3).

We assessed the goodness of fit of the model after grouping the patients according to their risk scores, which was a linear combination of the logarithms of tumor thickness and DCCD and of ulceration, into four groups (see Methods). We compared predicted and observed survival curves and found that Kaplan-Meier curves and predicted curves were superimposable over the complete range of disease courses for all four risk groups and that all four groups differed significantly from each other (Figure 7). Finally, we compared the goodness of fit for several models (a model based on Cox regression analysis, a model that includes data on Melan-A staining, and a model based on the current AJCC criteria). In summary, we found that the parametric model based on thickness, DCCD, and ulceration most accurately predicted melanoma death (Table S3).

## Discussion

In this study, we quantified the number of DCCs per one million isolated lymph node cells (DCCD) and assessed its utility in predicting melanoma outcome. Based on a median follow-up of 49 mo, with 370 patients having follow-up times of more than 5 y, we found that at the time of sentinel node biopsy, quantitative assessment of DCCD predicted melanoma outcome by univariable and multivariable analysis in a large cohort of patients. Furthermore, quantitative DCCD showed a stronger association with outcome than qualitative conventional histopathology and, when combined with primary melanoma thickness and ulceration, had a synergistic impact on patient survival. Using these variables we developed a parametric model that proved to be the most accurate for predicting outcome. Although we currently lack an external validation cohort with long follow-up, the accepted prognostic role of sentinel lymph node spread [4] and the successful internal validation (bootstrap and goodness of fit) give credibility to the findings.

We found that even the detection of three or fewer DCCs per million leukocytes in the sentinel node increases the risk of death at 5 y from melanoma by 6% (8% for DCCD = 0 versus 14% for 0< DCCD ≤3). This finding is in line with reports supporting the clinical relevance of single DCCs [5],[27] and the novel AJCC recommendation [4] to refrain from using a lower threshold for sentinel node spread. However, we also identified three shortcomings of the AJCC categorization approach. First, AJCC staging does not differentiate between isolated cancer cells and small and large microscopic metastases. Our data demonstrate that the number of cells matters over the full range of DCCD. Second, because any measured DCCD value can be translated into a 5-y survival rate, the typical exaggerations of categorizing staging systems, such as upgrading from stage II to stage III because of the detection of a single melanoma cell, are also avoided, and individual disease courses can be accommodated better than by categorizing tumors based on the AJCC staging system. Third, we provide clinical evidence for the context dependency of the metastasis-forming potential of DCCs, which emerges from our observation that DCCD, tumor thickness, and ulceration—being the leading prognostic factors from the multivariable analysis—can be combined in a parametric survival model where the prognostic value of a single DCC differs for thick and thin tumors. For example, we observed DCCs in 46% of T1 stage melanomas; however, 5-y survival rates are more than 90% in this subgroup of patients, indicating that under most conditions DCCs do not result in clinically relevant metastasis. This may suggest that cellular programs such as senescence or dormancy are activated at initial homing to distant sites [28],[29] but may be released once primary tumors grow large. Such a scenario has gained credibility since secreted factors of primary melanomas, such as exosomes, were shown to evoke substantial systemic effects [30] promoting metastasis.

In addition to DCCD and thickness of the primary melanoma, ulceration status had an impact on survival in melanoma. The biological interdependencies between destructive growth (ulceration), tumor-mass-induced systemic alterations (tumor thickness), and metastatic dissemination (DCCD) for progression of an individual melanoma may explain why histopathologically node-positive patients can have a better outcome than histopathologically node-negative patients if the primary melanoma has more favorable prognostic features. This phenomenon is not reflected within a categorizing staging system. For example, the current AJCC staging predicts a 5-y survival of 53% for stage IIC (T4bN0M0) and 70% for stage IIIa (T1-4N1aM0). In contrast, the estimated survival of a patient with tumor thickness 7.4 mm and DCCD = 0 but without ulceration is identical to the estimated survival of a patient with ulceration and tumor thickness 3.35 mm and DCCD = 2, or with tumor thickness 1.2 mm and DCCD = 772 in our model.

Since summary measures of survival may provide insufficient information about population dispersion, we asked whether the new model reflects the prognostic heterogeneity of patients more accurately. Indeed, we identified a group of patients at high risk for progression in whom the AJCC model underestimates the risk of death. Although this group of patients is relatively small (13% in our cohort), these patients will most likely benefit from adjuvant therapy, and the model may help to improve patient stratification for clinical trials. It also identified a group of very low risk patients who have an excellent long-term outcome and whose risk of dying is overestimated by the AJCC staging model.

We carefully evaluated the performance of our assay. As lymph node disaggregation destroys the tissue architecture, some morphological criteria to identify melanoma cells are lost. However, we deem it unlikely that benign nevus cells in sentinel nodes, described in up to 28% of melanoma patients [9], confound our conclusions. These cells rarely express gp100 [31], and likewise we could not find gp100-expressing cells in non-melanoma lymph nodes. While this does not rule out the possibility that truly DCC-negative sentinel nodes from melanoma patients may contain gp100-expressing benign nevus cells, our finding that even low numbers of gp100-positive cells are prognostically relevant would then suggest that gp100-positive nevus cells may be prognostically informative. Furthermore, in 97% of all analyzed gp100-positive cells we detected chromosomal or subchromosomal alterations. For only one patient out of 46 could we not confirm the malignant descent of the isolated cell. In all other cases genetic alterations in gp100-positive cells proved disseminated melanoma, suggesting that morphological criteria for DCC identification are dispensable.

Then, we directly addressed the question of whether staining for another antigen (Melan-A) increases the detection rate and the prognostic power of the gp100-based immunoassay. However, while Melan-A staining added a few samples (7%) to the gp100-identified positive lymph nodes, it also stained 8% of control nodes. Moreover, assessing the prognostic power of the combined results of gp100 and Melan-A staining for 710 patients, we found that the gp100-only model was more accurate.

Compared to our assay, evaluation of sentinel nodes by pathology has two major limitations. First, sensitivity largely depends on the number of slides examined. Second, quantification of lymphatic melanoma spread—a three-dimensional and often multilocular process—is impossible by histopathology. We resolved these problems by homogenizing the patchy spatial distribution of tumor cells within the node [32]—which greatly impacts detection in tissue sections but less so in our approach—and counting the stained cells. Thus, screening of a median of only two slides (2×10^6^ cells) per node revealed a detection rate of 51%, whereas pathology was positive in only 14% of patients. To achieve a similar sensitivity by histopathology, it has been suggested that more than 36 slides per sample need to be analyzed, indicating that immunocytology might be advantageous also for practical reasons [7],[12]. In the future, both lymph node preparation and screening may even be subjected to partial automation and thereby decrease workload further.

The high detection rate of immunocytology is reminiscent of the sensitivity of RT-PCR methods, which is also around 50% [33]. However, despite 20 y of clinical evaluation, RT-PCR assays have failed to become clinical routine. Since one of our major findings consists in the quantitative impact of lymphatic cancer cell dissemination for patient outcome, we deem the non-quantitative nature of RT-PCR assays and the failure to prove the malignant melanoma origin of the detected nucleic acids to be a likely explanation for its failure. RT-PCR assays do not measure cell numbers but transcript numbers, which may be generated by a few high-expressing cells or many low-expressing cells. Since the unit of selection during malignant progression is a cell and not a transcript, even quantitative transcript information will always represent a qualitative assessment of cancer spread. Therefore, RT-PCR methods are unable to provide the information delivered here that the prognostic weight of a single disseminated melanoma cell is context dependent.

In summary, we provide evidence that quantification of lymphatic cancer cell dissemination is feasible and can be combined with other quantitative and qualitative characteristics of the primary tumor for accurate individual outcome prediction, probably not only for melanoma but also for other types of solid cancer [34]. It will be important to validate the findings in an independent study before the assay and the prediction model are used clinically.

## Supporting Information

Figure S1 **gp100 and Melan-A double staining.** Detection of a double-positive melanoma cell in the sentinel lymph node (gp100/HMB45, green; Melan-A, red; nuclear DAPI, blue). The right panel shows an overlay of the three images.
(TIF)

Figure S2 **Prognostic factors and melanoma survival.** The Kaplan-Meier curves for the standard prognostic factors and grouped DCCDs.
(TIF)

Figure S3 **Hazard ratios of tumor thickness, DCCD, and ulceration over time for Cox and lognormal models.** The ratio of two cumulative hazard functions is plotted as a function of follow-up time for the three predictive variables DCCD, tumor thickness, and ulceration. For DCCD we compared DCCD >0 with DCCD = 0. For tumor thickness we compared tumor thickness >1.85 mm ( = median tumor thickness) with tumor thickness ≤1.85 mm. For ulceration we compared positive with negative outcome. For each of the three variables we calculated the empirical ratios of the cumulative hazard functions as −log(Kaplan-Meier survival), depicted as green step functions. The blue lines show the constant hazard ratios obtained from the corresponding Cox models. The red curves show the ratios of the cumulative hazard functions of the corresponding lognormal parametric survival model. There is clear evidence for DCCD and tumor thickness that the observed ratios of the cumulative hazard functions depart from a constant value. They show a steady decline over the whole follow-up period of more than 10 y.
(TIF)

Figure S4 **Synergism between tumor thickness and DCCD for outcome prediction.** 5-y survival probability (percent) as a function of tumor thickness and DCCD for all patients without ulceration (blue isoboles) and with ulceration (red isoboles) on a linear scale. Convex isoboles indicate synergism.
(TIF)

Table S1 **DCCD in relation to standard prognostic factors in 1,027 patients.**
(DOCX)

Table S2 **Observed and expected number of deaths for three groups of patients at the time of follow-up for each patient.**
(DOCX)

Table S3 **Goodness of fit of four multivariable survival models.**
(DOCX)

Text S1 **Model equations.**
(DOC)

## Abbreviations

AJCC: American Joint Committee on Cancer
BIC: Bayes information criterion
CGH: comparative genomic hybridization
DCC: disseminated cancer cell
DCCD: disseminated cancer cell density
RT-PCR: reverse transcriptase polymerase chain reaction
