# Supplementary figures and images for: Quantitative Measurement of Melanoma Spread in Sentinel Lymph Nodes and Survival
Source: PLoS Med. 2014 Feb 18;11(2):e1001604. doi: 10.1371/journal.pmed.1001604 (PMC3928050; doi:10.1371/journal.pmed.1001604)

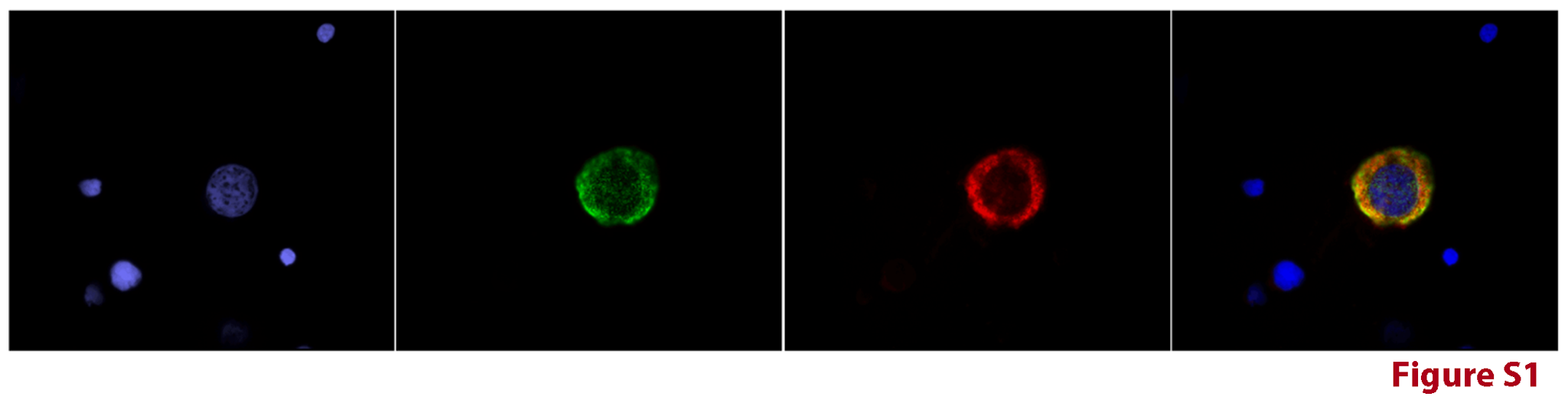

Supplement: Figure S1 — gp100 and Melan-A double staining. Detection of a double-positive melanoma cell in the sentinel lymph node (gp100/HMB45, green; Melan-A, red; nuclear DAPI, blue). The right panel shows an overlay of the three images. (TIF) [file pmed.1001604.s001.tif]

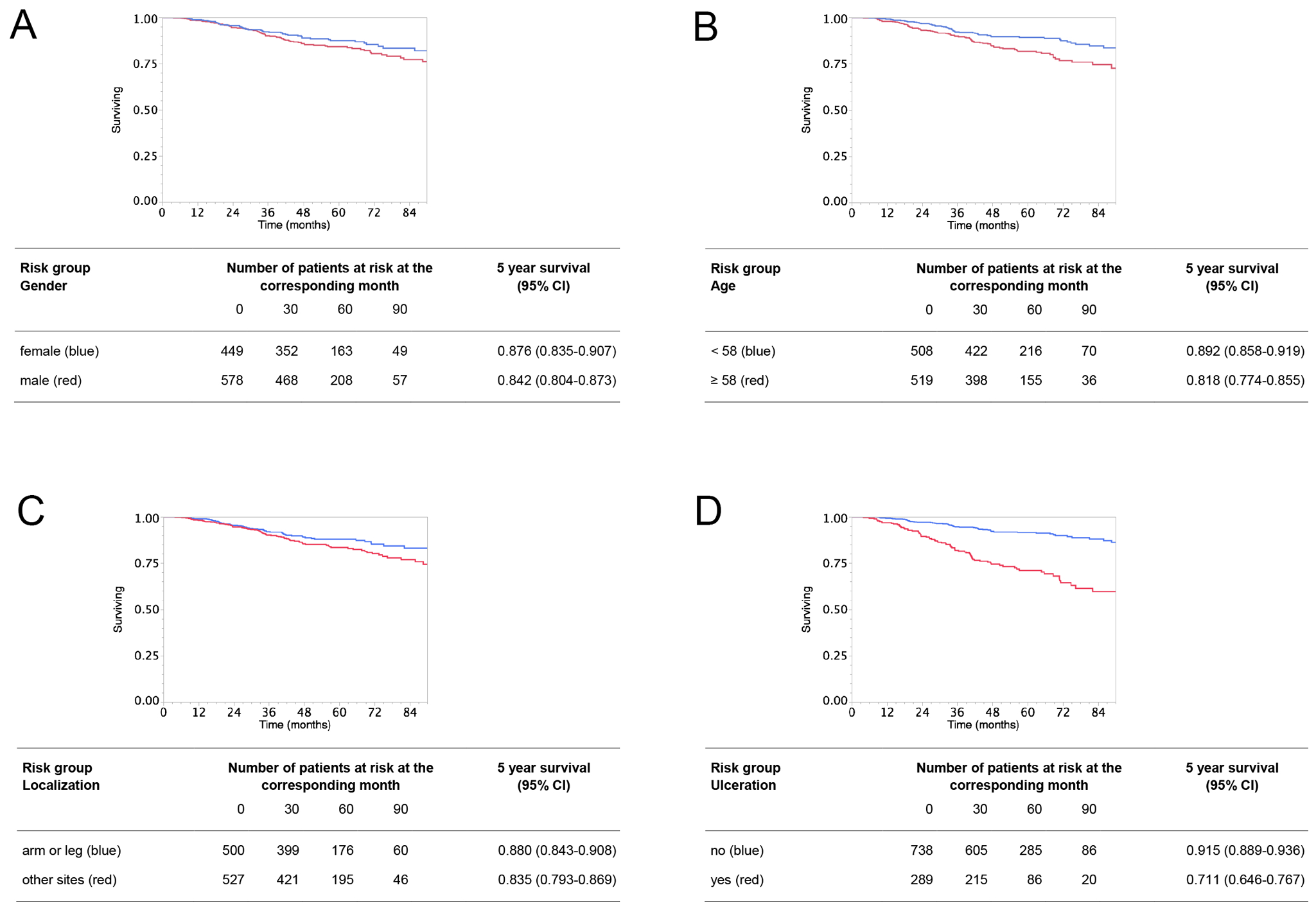

Supplement: Figure S2 — Prognostic factors and melanoma survival. The Kaplan-Meier curves for the standard prognostic factors and grouped DCCDs. (TIF) [file pmed.1001604.s002.tif]

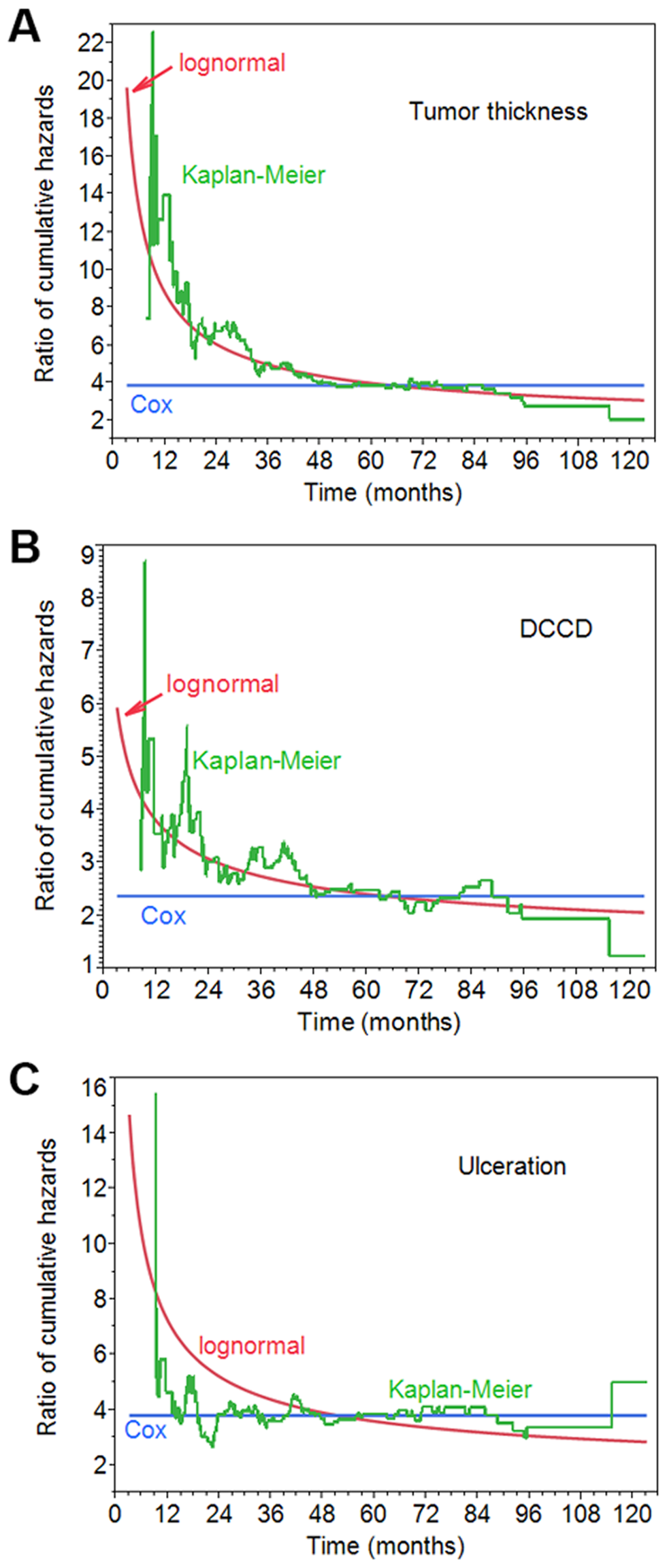

Supplement: Figure S3 — Hazard ratios of tumor thickness, DCCD, and ulceration over time for Cox and lognormal models. The ratio of two cumulative hazard functions is plotted as a function of follow-up time for the three predictive variables DCCD, tumor thickness, and ulceration. For DCCD we compared DCCD >0 with DCCD = 0. For tumor thickness we compared tumor thickness >1.85 mm ( = median tumor thickness) with tumor thickness ≤1.85 mm. For ulceration we compared positive with negative outcome. For each of the three variables we calculated the empirical ratios of the cumulative hazard functions as −log(Kaplan-Meier survival), depicted as green step functions. The blue lines show the constant hazard ratios obtained from the corresponding Cox models. The red curves show the ratios of the cumulative hazard functions of the corresponding lognormal parametric survival model. There is clear evidence for DCCD and tumor thickness that the observed ratios of the cumulative hazard functions depart from a constant value. They show a steady decline over the whole follow-up period of more than 10 y. (TIF) [file pmed.1001604.s003.tif]

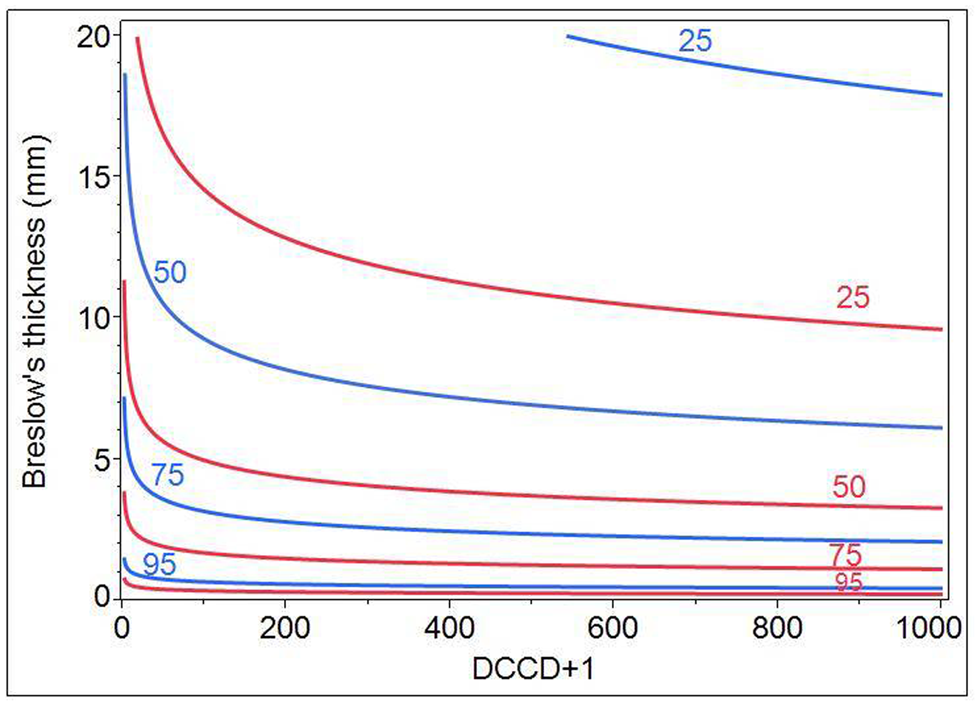

Supplement: Figure S4 — Synergism between tumor thickness and DCCD for outcome prediction. 5-y survival probability (percent) as a function of tumor thickness and DCCD for all patients without ulceration (blue isoboles) and with ulceration (red isoboles) on a linear scale. Convex isoboles indicate synergism. (TIF) [file pmed.1001604.s004.tif]
