# Supplementary material for: Quantitative Measurement of Melanoma Spread in Sentinel Lymph Nodes and Survival
Source: PLoS Med. 2014 Feb 18;11(2):e1001604. doi: 10.1371/journal.pmed.1001604 (PMC3928050; doi:10.1371/journal.pmed.1001604)
Supplement: Table S1 — DCCD in relation to standard prognostic factors in 1,027 patients. (DOCX) [file pmed.1001604.s005.docx]

**Supplementary Table 1. DCCD in relation to standard prognostic factors in 1027 patients.**

| Variable |  | Number of DCC negative patients | Number of DCC positive patients | p-value* |
| --- | --- | --- | --- | --- |
| Gender | |  |  |  |
| male | | 266 (46%) | 312 (54%) | 0.04 |
| female | | 236 (53%) | 213 (47%) |  |
| Age | |  |  |  |
| < 58 | | 244 (48%) | 264 (52%) | 0.60 |
| ≥ 58 | | 258 (50%) | 261 (50%) |  |
| Ulceration | |  |  |  |
| yes | | 123 (43%) | 166 (57%) | 0.01 |
| no | | 379 (51%) | 359 (49%) |  |
| Localization | |  |  |  |
| arm or leg | | 258 (52%) | 242 (48%) | 0.09 |
| other sites | | 244 (46%) | 283 (54%) |  |
| Nodal status (pathology) | |  |  |  |
| negative | | 483 (54%) | 405 (46%) | <0.0001 |
| positive | | 19 (14%) | 120 (86%) |  |
| Thickness** | |  |  |  |
| T1 | | 45 (54%) | 38 (46%) | 0.004† |
| T2 | | 261 (53%) | 235 (47%) |  |
| T3 | | 142 (45%) | 173 (55%) |  |
| T4 | | 54 (41%) | 79 (59%) |  |

* Pearson chi-square test if not indicated otherwise

** T-stages according to the TNM classification,

† logistic regression analysis: test for trend
