# Supplementary material for: Quantitative Measurement of Melanoma Spread in Sentinel Lymph Nodes and Survival
Source: PLoS Med. 2014 Feb 18;11(2):e1001604. doi: 10.1371/journal.pmed.1001604 (PMC3928050; doi:10.1371/journal.pmed.1001604)
Supplement: Table S2 — Observed and expected number of deaths for three groups of patients at the time of follow-up for each patient. (DOCX) [file pmed.1001604.s006.docx]

**Supplementary Table 2. Observed and expected number of deaths for three groups of patients at the time of follow-up for each patient.**

| **Group** | **N Patients** | **Exp Deaths AJCC** | **Exp Deaths New Model** | **Obs deaths** | **Chi sq**  **AJCC** | **Chi sq New Model** | **P-value New Model** | **P-value AJCC** |
| --- | --- | --- | --- | --- | --- | --- | --- | --- |
| S1 | 124 | 32.6 | 17.7 | 25 | 1.8 | 3.0 | 0.0832 | 0.1824 |
| S2 | 136 | 22.7 | 36.9 | 43 | 18.1 | 1.0 | 0.3180 | <.0001* |
| S3 | 767 | 63.2 | 61.6 | 70 | 0.7 | 1.1 | 0.2863 | 0.3926 |

Please, note that for Group S1 and Group S3 both models provide acceptable fit. We therefore combined Group S1 and Group S3 to a novel Group 1 for Figure 6.
