# Supplementary material for: Quantitative Measurement of Melanoma Spread in Sentinel Lymph Nodes and Survival
Source: PLoS Med. 2014 Feb 18;11(2):e1001604. doi: 10.1371/journal.pmed.1001604 (PMC3928050; doi:10.1371/journal.pmed.1001604)
Supplement: Table S3 — Goodness of fit of four multivariable survival models. (DOCX) [file pmed.1001604.s007.docx]

**Supplementary Table 3. Goodness-of-fit of four multivariable survival models.**


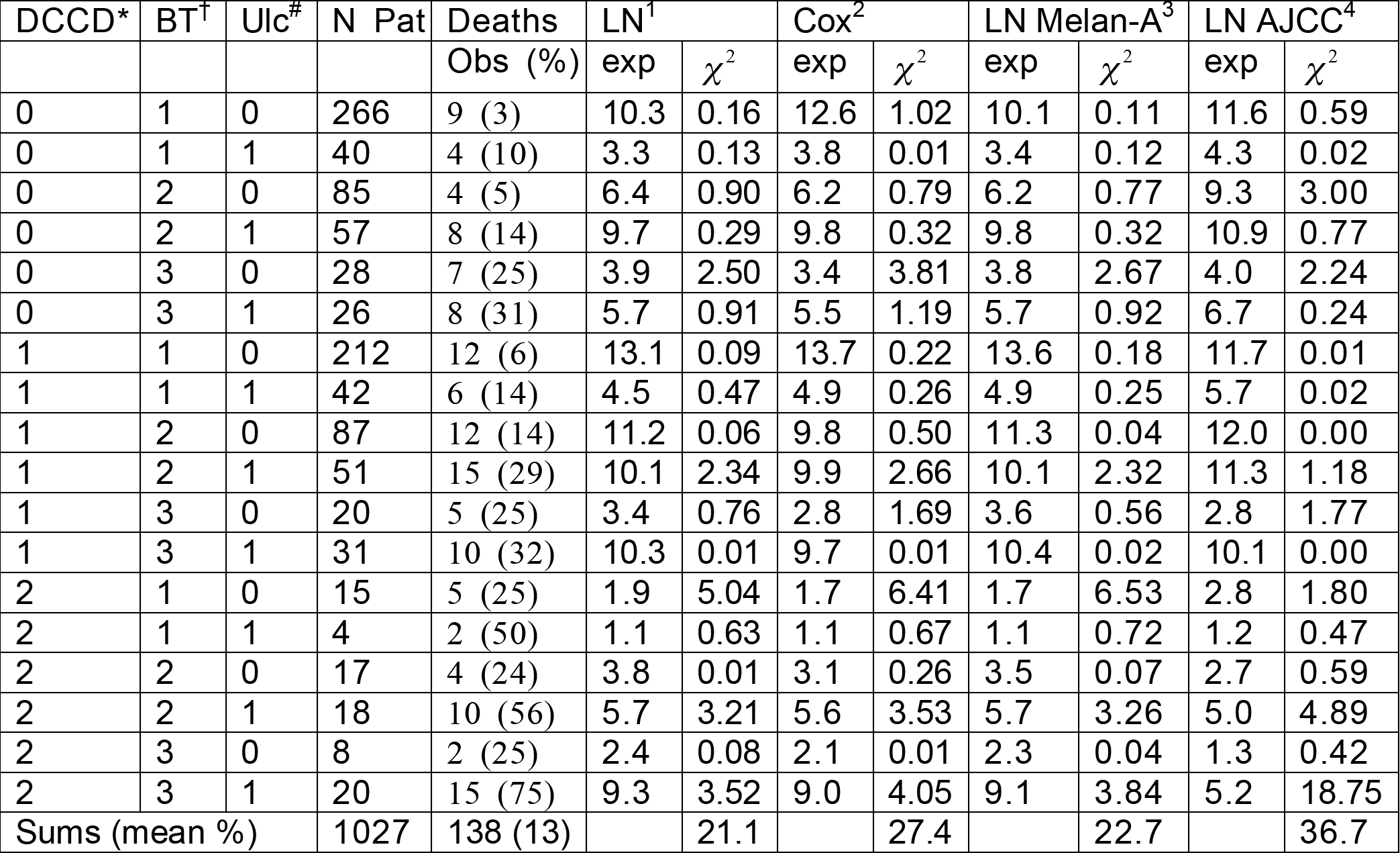


* DCCD = disseminated cancer cell density. In contrast to Table 2 in the manuscript, the patients were divided into only three subgroups: 0 (DCCD=0), 1 (0<DCCD<100, with a median of 3), and 2 (100≤DCCD, with a median of 1081).

**†** BT = Breslow’s thickness; 1 (BT≤2 mm), 2 (2 mm<BT≤4 mm), 3 (4 mm<BT)

# Ulc = Ulceration; 0: no, 1: yes

^1^ The lognormal model LN includes DCCD, Breslow’s thickness (*BT*) and ulceration (*Ulc*).; p=0.13

^2^ The “Cox” model includes DCCD, Breslow’s thickness (*BT*) and ulceration (*Ulc*).; p=0.03

^3^ The “LN Melan-A” model includes the maximum of DCCD (gp100) and DCCD (Melan-A), Breslow’s thickness (*BT*) and ulceration (Ulc). p=0.09

^4^ The “LN AJCC” model is based on the staging according to the AJCC; p<0.0002
