# Supplementary material for: Quantitative Measurement of Melanoma Spread in Sentinel Lymph Nodes and Survival
Source: PLoS Med. 2014 Feb 18;11(2):e1001604. doi: 10.1371/journal.pmed.1001604 (PMC3928050; doi:10.1371/journal.pmed.1001604)
Supplement: Text S1 — Model equations. (DOC) [file pmed.1001604.s008.doc]

**Supplementary text S1: Model equations**

For the lognormal model involving DCCD, tumor thickness and ulceration the probability to survive up to time $t$ equals

$$1-\Phi\left(\frac{\ln t-\left(6.25-0.29\log(DCCD+1)-1.62\log(BT)-0.44Ulc\right)}{1.12}\right),$$

where log refers here and in the following formulas to the decadic logarithm and ln to the natural logarithm. The function $\Phi$ is the cumulative distribution function of the standard normal distribution.

Melan-A was analyzed in the 710 patients for whom additional slides were available. The probability to survive up to time $t$equals

$$1-\Phi\left(\frac{\ln t-\left(6.20-0.27\log[\max(DCCD+1,Melan\text{-}A+1)]-1.63\log(BT)-0.46Ulc\right)}{1.13}\right).$$

The “AJCC” model is based on the staging according to the AJCC and includes the pathological status of the sentinel node, Breslow’s thickness and ulceration. The probability to survive up to time $t$equals

$$1-\Phi\left(\frac{\ln t-(6.87-0.80"IB"-1.48"IIA"-1.82"IIB"-2.42"IIC"-2.10"IIIA"-2.79"IIIB")}{1.18}\right).$$
